# 6-[4-(*tert*-Butyl­dimethyl­sil­yloxy)phen­yl]-1-oxa­spiro­[2.5]hepta­ne

**DOI:** 10.1107/S241431462400590X

**Published:** 2024-06-21

**Authors:** Edward A. Wetzel, Sergey Lindeman, William A. Donaldson

**Affiliations:** a Department of Chemistry Marquette University, P O Box 1881 Milwaukee WI 53201-1881 USA; University of Aberdeen, United Kingdom

**Keywords:** crystal structure, organic, spiro­epoxide

## Abstract

The title compound, C_19_H_30_O_2_Si, has triclinic (*P*) symmetry at 100 K. The O atom of the epoxide group has a pseudoaxial orientation and the dihedral angle between the cyclo­hexyl and benzene rings is 85.80 (8)°. The C—O—Si—C_*t*_ (*t* = *tert*-but­yl) torsion angle is −177.40 (14)°. In the crystal, pairwise C—H⋯O links connect the mol­ecules into inversion dimers featuring *R*^2^_2_(8) loops.

## Abstract 2

In the title compound, the O atom of the epoxide group has a pseudoaxial orientation and the dihedral angle between the cyclo­hexyl and benzene rings is 85.80 (8)°. The C—O—Si—C_*t*_ (*t* = *tert*-but­yl) torsion angle is −177.40 (14)°. In the crystal, pairwise C—H⋯O links connect the mol­ecules into inversion dimers featuring 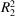
(8) loops.

## Graphical abstract

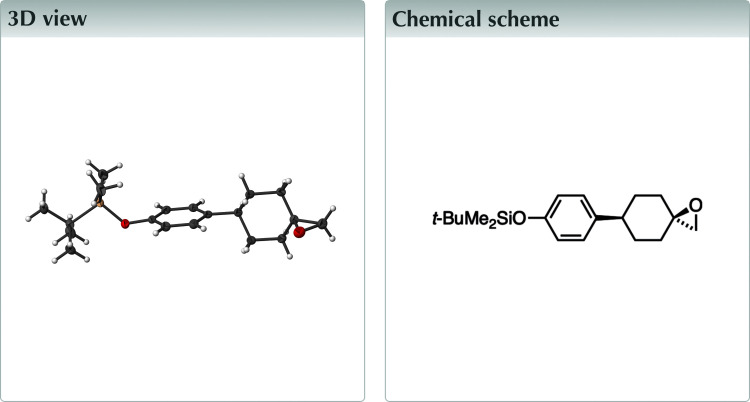

## Structure description

Fumagillin and ovalicin, isolated from *Aspergillus fumigatus* and *Pseudorotium ovalis*, respectively, are sesquiterpene epoxides that exhibit anti-angiogenic activity. The key structural feature of both is a 1-oxo­spiro­[2.5]heptane moiety. The structure of fumagillin was initially deduced by X-ray crystallographic analysis of its hydrolysis product fumagillol (McCorkindale & Sime, 1961 ▸) and the X-ray crystal structure of fumagillin was eventually reported (Halasz *et al.*, 2000 ▸). Preparation of the 1-oxo­spiro­[2.5]heptane system by reaction of di­methyl­sulfoxonium methyl­ide with substituted cyclo­hexa­nones generally proceeds with the formation of the exocyclic epoxide in which the oxygen atom has an axial orientation (Corey & Chaykovsky, 1965 ▸; Carlson & Behn, 1967 ▸). In connection with our studies on estrogen receptor beta-selective agonists (Hanson *et al.*, 2018 ▸; Wetzel *et al.*, 2022 ▸), we had the opportunity to prepare the title compound, C_19_H_30_O_2_Si, and we now present its synthesis and crystal structure.

**Figure 1:**
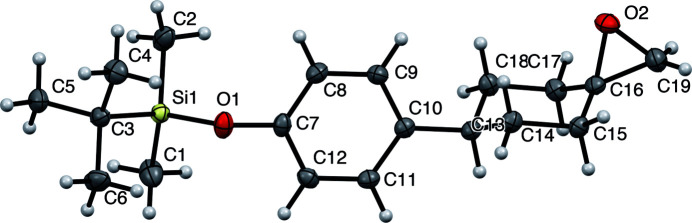
The mol­ecular structure of the title compound showing 50% displacement ellipsoids.

**Figure 2:**
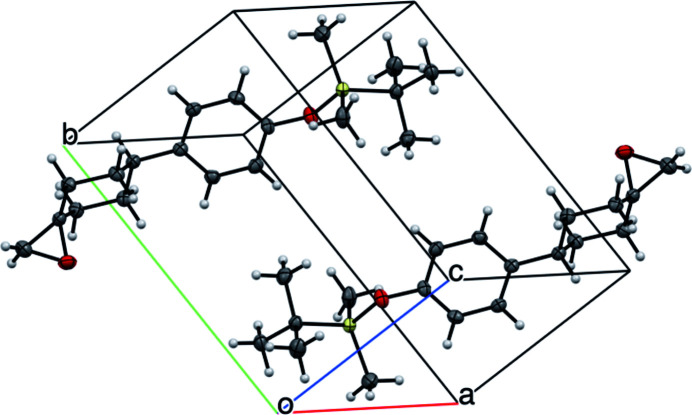
Unit-cell packing of the title compound.

The title compound (Fig. 1 ▸) has an extended conformation with a *transoid t*-Bu—Si—O—Ar moiety. The cyclo­hexane ring has a chair conformation with the aryl substituent in an equatorial position and the epoxide oxygen atom in a pseudoaxial orientation. The C7—O1—Si1—C3 torsion angle is −177.40 (14)°. In the crystal, a weak C—H⋯O link (Fig. 2 ▸, Table 1 ▸) connects the mol­ecules into inversion dimers featuring 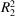
(8) loops.

## Synthesis and crystallization

**Figure 3:**
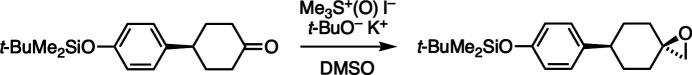
Reaction scheme.

The reaction scheme is shown in Fig. 3 ▸. To a solution of potassium *tert*-butoxide (2.317 g, 20.65 mmol) in di­methyl­sulfoxide (DMSO) (10 ml) was added tri­methyl­sulfoxonium iodide (5.00 g, 22.72 mmol). The solution was stirred for 30 min, and then a solution of 4-[(4-*t*-butyl­dimethyl­sil­yloxy)phen­yl]cyclo­hexa­none (6.288 g, 20.65 mmol) in DMSO (50 ml) was added dropwise. The mixture was stirred for 24 h, and then partitioned between ethyl acetate and water. The aqueous layer was extracted with ethyl acetate. The combined organic extracts were washed with brine, dried (MgSO_4_), and concentrated. Upon standing at room temperature overnight, colorless crystals formed. The crystals were filtered off to afford the title compound (5.262 g, 80%). Recrystallization from the mixed solvents of ethyl acetate/hexa­nes gave colorless flat prisms. ^1^H NMR (400 MHz, CDCl_3_) δ 7.08 (*d*, *J* = 8.0 Hz, 2H), 6.76 (*d*, *J* = 8.0 Hz, 2H), 2.68 (*s*, 2H), 2.54 (*t*, *J* = 8.2 Hz, 1H), 2.10–1.97 (*m*, 2H), 1.92–1.74 (*m*, 4H), 1.35 (*d*, *J* = Hz, 2H), 0.97 (*s*, 9H), 0.18 (*s*, 6H); ^13^C NMR (100 MHz, CDCl_3_) δ 153.7, 139.3, 127.6, 119.8, 57.9, 53.9, 42.4, 33.2, 31.7, 25.6, 18.1, −4.4 p.p.m..

## Refinement

Crystal data, data collection and structure refinement details are summarized in Table 2 ▸.

## Supplementary Material

Crystal structure: contains datablock(s) global, I. DOI: 10.1107/S241431462400590X/hb4478sup1.cif

Structure factors: contains datablock(s) I. DOI: 10.1107/S241431462400590X/hb4478Isup2.hkl

Supporting information file. DOI: 10.1107/S241431462400590X/hb4478Isup3.cml

CCDC reference: 2363391

Additional supporting information:  crystallographic information; 3D view; checkCIF report

## Figures and Tables

**Table 1:** Hydrogen-bond geometry (Å, °)

*D*—H⋯*A*	*D*—H	H⋯*A*	*D*⋯*A*	*D*—H⋯*A*
C15—H15*A*⋯O2^i^	0.99	2.54	3.487 (2)	159

Symmetry code: (i) 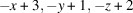.

**Table 2:** Experimental details

Crystal data	
Chemical formula	C_19_H_30_O_2_Si
*M*_r_	318.52
Crystal system, space group	Triclinic, *P* 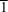
Temperature (K)	100
*a*, *b*, *c* (Å)	7.3673 (3), 11.2285 (4), 12.5778 (5)
α, β, γ (°)	104.974 (4), 101.964 (4), 103.460 (4)
*V* (Å^3^)	937.09 (7)
*Z*	2
Radiation type	Cu *K*α
μ (mm^−1^)	1.13
Crystal size (mm)	0.74 × 0.43 × 0.07

Data collection	
Diffractometer	Rigaku Oxford Diffraction SuperNova, Dual, Cu at home/near, Atlas
Absorption correction	Gaussian (*CrysAlis PRO*; Rigaku OD, 2018 ▸)
*T*_min_, *T*_max_	0.184, 1.000
No. of measured, independent and observed [*I* > 2σ(*I*)] reflections	16772, 3539, 3276
*R*_int_	0.046
(sin θ/λ)_max_ (Å^−1^)	0.612

Refinement	
*R*[*F*^2^ > 2σ(*F*^2^)], *wR*(*F*^2^), *S*	0.046, 0.131, 1.10
No. of reflections	3539
No. of parameters	212
H-atom treatment	H atoms treated by a mixture of independent and constrained refinement
Δρ_max_, Δρ_min_ (e Å^−3^)	0.70, −0.35

Computer programs: *CrysAlis PRO* (Rigaku OD, 2018 ▸), *OLEX2.solve* (Bourhis *et al.*, 2015 ▸), *SHELXL* (Sheldrick, 2015 ▸), and *OLEX2* (Dolomanov *et al.*, 2009 ▸).

## full crystallographic data

### 6-[4-(tert-Butyldimethylsilyloxy)phenyl]-1-oxaspiro[2.5]heptane Crystal data

**Table:**

C_19_H_30_O_2_Si	*Z* = 2
*M**_r_* = 318.52	*F*(000) = 348
Triclinic, *P*1	*D*_x_ = 1.129 Mg m^−^^3^
*a* = 7.3673 (3) Å	Cu *K*α radiation, λ = 1.54184 Å
*b* = 11.2285 (4) Å	Cell parameters from 9134 reflections
*c* = 12.5778 (5) Å	θ = 4.3–70.5°
α = 104.974 (4)°	µ = 1.13 mm^−^^1^
β = 101.964 (4)°	*T* = 100 K
γ = 103.460 (4)°	Plate, colourless
*V* = 937.09 (7) Å^3^	0.74 × 0.43 × 0.07 mm

### 6-[4-(tert-Butyldimethylsilyloxy)phenyl]-1-oxaspiro[2.5]heptane Data collection

**Table:**

Rigaku Oxford Diffraction SuperNova, Dual, Cu at home/near, Atlas diffractometer	3539 independent reflections
Radiation source: micro-focus sealed X-ray tube, SuperNova (Cu) X-ray Source	3276 reflections with *I* > 2σ(*I*)
Mirror monochromator	*R*_int_ = 0.046
Detector resolution: 10.3756 pixels mm^-1^	θ_max_ = 70.6°, θ_min_ = 3.8°
ω scans	*h* = −8→8
Absorption correction: gaussian (CrysAlisPro; Rigaku OD, 2018)	*k* = −13→13
*T*_min_ = 0.184, *T*_max_ = 1.000	*l* = −15→15
16772 measured reflections	

### 6-[4-(tert-Butyldimethylsilyloxy)phenyl]-1-oxaspiro[2.5]heptane Refinement

**Table:**

Refinement on *F*^2^	Primary atom site location: iterative
Least-squares matrix: full	Hydrogen site location: mixed
*R*[*F*^2^ > 2σ(*F*^2^)] = 0.046	H atoms treated by a mixture of independent and constrained refinement
*wR*(*F*^2^) = 0.131	*w* = 1/[σ^2^(*F*_o_^2^) + (0.0862*P*)^2^ + 0.2247*P*] where *P* = (*F*_o_^2^ + 2*F*_c_^2^)/3
*S* = 1.10	(Δ/σ)_max_ < 0.001
3539 reflections	Δρ_max_ = 0.70 e Å^−^^3^
212 parameters	Δρ_min_ = −0.35 e Å^−^^3^
0 restraints	

### 6-[4-(tert-Butyldimethylsilyloxy)phenyl]-1-oxaspiro[2.5]heptane Special details

**Table:**

Geometry. All esds (except the esd in the dihedral angle between two l.s. planes) are estimated using the full covariance matrix. The cell esds are taken into account individually in the estimation of esds in distances, angles and torsion angles; correlations between esds in cell parameters are only used when they are defined by crystal symmetry. An approximate (isotropic) treatment of cell esds is used for estimating esds involving l.s. planes.

### 6-[4-(tert-Butyldimethylsilyloxy)phenyl]-1-oxaspiro[2.5]heptane Fractional atomic coordinates and isotropic or equivalent isotropic displacement parameters (Å^2^)

**Table:**

	*x*	*y*	*z*	*U*_iso_*/*U*_eq_	
Si1	0.40119 (5)	0.19395 (4)	0.24021 (3)	0.01921 (16)	
O1	0.47620 (15)	0.22061 (11)	0.38036 (9)	0.0263 (3)	
O2	1.64517 (17)	0.47505 (10)	0.88309 (10)	0.0292 (3)	
C1	0.3887 (3)	0.02593 (17)	0.16096 (17)	0.0356 (4)	
H1A	0.518022	0.014659	0.180830	0.053*	
H1B	0.344470	0.010513	0.078168	0.053*	
H1C	0.296937	−0.035756	0.182195	0.053*	
C2	0.5762 (2)	0.31022 (18)	0.20005 (16)	0.0315 (4)	
H2A	0.556202	0.395409	0.222952	0.047*	
H2B	0.555172	0.279906	0.116802	0.047*	
H2C	0.709501	0.316690	0.239220	0.047*	
C3	0.1549 (2)	0.21993 (14)	0.21951 (13)	0.0220 (3)	
C4	0.1760 (2)	0.35451 (16)	0.29980 (15)	0.0290 (4)	
H4A	0.228450	0.359582	0.379644	0.044*	
H4B	0.048251	0.368942	0.289519	0.044*	
H4C	0.264834	0.420774	0.281404	0.044*	
C5	0.0722 (2)	0.21241 (16)	0.09442 (15)	0.0287 (4)	
H5A	0.156028	0.282682	0.077753	0.043*	
H5B	−0.059390	0.220714	0.083054	0.043*	
H5C	0.067239	0.129025	0.042720	0.043*	
C6	0.0151 (2)	0.11714 (17)	0.24914 (17)	0.0327 (4)	
H6A	0.000713	0.030972	0.198313	0.049*	
H6B	−0.112221	0.132184	0.238917	0.049*	
H6C	0.067749	0.122664	0.329095	0.049*	
C7	0.6508 (2)	0.21980 (15)	0.44469 (12)	0.0208 (3)	
C8	0.8018 (2)	0.33469 (14)	0.50020 (13)	0.0237 (3)	
H8	0.788337	0.412995	0.488878	0.028*	
C9	0.9723 (2)	0.33456 (14)	0.57228 (13)	0.0226 (3)	
H9	1.075023	0.413377	0.609708	0.027*	
C10	0.9962 (2)	0.22092 (14)	0.59095 (12)	0.0196 (3)	
C11	0.8430 (2)	0.10703 (14)	0.53377 (13)	0.0212 (3)	
H11	0.855594	0.028445	0.544784	0.025*	
C12	0.6725 (2)	0.10593 (14)	0.46116 (13)	0.0222 (3)	
H12	0.570384	0.027005	0.422645	0.027*	
C13	1.1790 (2)	0.22081 (13)	0.67256 (12)	0.0200 (3)	
H13	1.167369	0.128847	0.667431	0.024*	
C14	1.1985 (2)	0.29503 (15)	0.79754 (13)	0.0228 (3)	
H14A	1.206850	0.386201	0.804842	0.027*	
H14B	1.080957	0.256789	0.818228	0.027*	
C15	1.3792 (2)	0.29093 (15)	0.88093 (13)	0.0230 (3)	
H15A	1.394933	0.347476	0.959145	0.028*	
H15B	1.361595	0.201524	0.883051	0.028*	
C16	1.5596 (2)	0.33507 (13)	0.84517 (13)	0.0214 (3)	
C17	1.5456 (2)	0.26890 (15)	0.72234 (13)	0.0232 (3)	
H17A	1.539907	0.177436	0.711453	0.028*	
H17B	1.663369	0.311268	0.703953	0.028*	
C18	1.3644 (2)	0.27461 (14)	0.64068 (13)	0.0225 (3)	
H18A	1.352443	0.223818	0.561100	0.027*	
H18B	1.378788	0.365426	0.643765	0.027*	
C19	1.7490 (2)	0.39772 (16)	0.93104 (15)	0.0285 (4)	
H19A	1.865 (3)	0.394 (2)	0.9095 (18)	0.031 (5)*	
H19B	1.758 (3)	0.4120 (18)	1.0091 (18)	0.024 (4)*	

### 6-[4-(tert-Butyldimethylsilyloxy)phenyl]-1-oxaspiro[2.5]heptane Atomic displacement parameters (Å^2^)

**Table:**

	*U*^11^	*U*^22^	*U*^33^	*U*^12^	*U*^13^	*U*^23^
Si1	0.0177 (2)	0.0210 (2)	0.0203 (3)	0.00703 (16)	0.00510 (16)	0.00808 (17)
O1	0.0212 (5)	0.0382 (6)	0.0223 (6)	0.0115 (5)	0.0069 (4)	0.0116 (5)
O2	0.0337 (6)	0.0184 (5)	0.0308 (6)	0.0041 (4)	0.0067 (5)	0.0055 (4)
C1	0.0330 (9)	0.0308 (9)	0.0370 (10)	0.0150 (7)	0.0022 (7)	0.0023 (7)
C2	0.0219 (8)	0.0424 (9)	0.0390 (10)	0.0118 (7)	0.0103 (7)	0.0245 (8)
C3	0.0176 (7)	0.0226 (7)	0.0274 (8)	0.0061 (5)	0.0066 (6)	0.0105 (6)
C4	0.0250 (8)	0.0288 (8)	0.0356 (9)	0.0123 (6)	0.0108 (7)	0.0089 (7)
C5	0.0227 (8)	0.0300 (8)	0.0316 (9)	0.0079 (6)	0.0020 (6)	0.0114 (7)
C6	0.0223 (8)	0.0334 (9)	0.0483 (11)	0.0068 (6)	0.0133 (7)	0.0220 (8)
C7	0.0196 (7)	0.0285 (7)	0.0164 (7)	0.0093 (6)	0.0062 (5)	0.0082 (6)
C8	0.0279 (8)	0.0219 (7)	0.0247 (8)	0.0096 (6)	0.0083 (6)	0.0105 (6)
C9	0.0226 (7)	0.0199 (7)	0.0234 (8)	0.0034 (5)	0.0053 (6)	0.0072 (6)
C10	0.0213 (7)	0.0212 (7)	0.0179 (7)	0.0072 (5)	0.0082 (6)	0.0064 (6)
C11	0.0242 (7)	0.0186 (7)	0.0226 (7)	0.0071 (5)	0.0089 (6)	0.0072 (6)
C12	0.0217 (7)	0.0220 (7)	0.0201 (7)	0.0032 (5)	0.0070 (6)	0.0043 (6)
C13	0.0211 (7)	0.0194 (7)	0.0206 (7)	0.0069 (5)	0.0066 (6)	0.0071 (6)
C14	0.0231 (7)	0.0267 (7)	0.0216 (8)	0.0092 (6)	0.0090 (6)	0.0090 (6)
C15	0.0252 (8)	0.0254 (7)	0.0200 (7)	0.0082 (6)	0.0072 (6)	0.0088 (6)
C16	0.0237 (7)	0.0183 (7)	0.0225 (8)	0.0072 (5)	0.0053 (6)	0.0073 (6)
C17	0.0209 (7)	0.0249 (7)	0.0230 (8)	0.0072 (6)	0.0073 (6)	0.0054 (6)
C18	0.0219 (7)	0.0270 (7)	0.0183 (7)	0.0065 (6)	0.0075 (6)	0.0063 (6)
C19	0.0250 (8)	0.0306 (8)	0.0254 (9)	0.0058 (6)	0.0040 (6)	0.0068 (7)

### 6-[4-(tert-Butyldimethylsilyloxy)phenyl]-1-oxaspiro[2.5]heptane Geometric parameters (Å, º)

**Table:**

Si1—O1	1.6580 (11)	C8—C9	1.389 (2)
Si1—C1	1.8629 (17)	C9—C10	1.399 (2)
Si1—C2	1.8570 (16)	C10—C11	1.394 (2)
Si1—C3	1.8804 (15)	C10—C13	1.515 (2)
O1—C7	1.3735 (18)	C11—C12	1.387 (2)
O2—C16	1.4567 (17)	C13—C14	1.536 (2)
O2—C19	1.446 (2)	C13—C18	1.536 (2)
C3—C4	1.538 (2)	C14—C15	1.533 (2)
C3—C5	1.537 (2)	C15—C16	1.507 (2)
C3—C6	1.536 (2)	C16—C17	1.502 (2)
C7—C8	1.391 (2)	C16—C19	1.461 (2)
C7—C12	1.386 (2)	C17—C18	1.532 (2)
			
O1—Si1—C1	109.67 (8)	C9—C10—C13	121.75 (13)
O1—Si1—C2	109.22 (7)	C11—C10—C9	117.59 (14)
O1—Si1—C3	103.07 (6)	C11—C10—C13	120.64 (13)
C1—Si1—C3	112.59 (8)	C12—C11—C10	121.44 (14)
C2—Si1—C1	109.20 (9)	C7—C12—C11	120.11 (14)
C2—Si1—C3	112.88 (7)	C10—C13—C14	111.57 (12)
C7—O1—Si1	128.73 (9)	C10—C13—C18	112.72 (12)
C19—O2—C16	60.42 (10)	C18—C13—C14	110.02 (12)
C4—C3—Si1	108.90 (10)	C15—C14—C13	111.77 (12)
C5—C3—Si1	109.90 (10)	C16—C15—C14	111.09 (12)
C5—C3—C4	109.25 (13)	O2—C16—C15	113.99 (12)
C6—C3—Si1	110.17 (10)	O2—C16—C17	114.06 (12)
C6—C3—C4	108.91 (13)	O2—C16—C19	59.43 (10)
C6—C3—C5	109.69 (13)	C17—C16—C15	115.08 (12)
O1—C7—C8	120.15 (13)	C19—C16—C15	120.42 (14)
O1—C7—C12	120.07 (13)	C19—C16—C17	120.66 (13)
C12—C7—C8	119.63 (14)	C16—C17—C18	111.04 (12)
C9—C8—C7	119.77 (14)	C17—C18—C13	111.41 (12)
C8—C9—C10	121.45 (14)	O2—C19—C16	60.15 (10)
			
O1—Si1—C3—C4	54.6 (1)	C8—C7—C12—C11	0.8 (2)
O1—Si1—C3—C5	174.2 (1)	C7—C12—C11—C10	−0.5 (2)
O1—Si1—C3—C6	−64.8 (1)	C9—C10—C13—C14	66.7 (2)
O1—C7—C8—C9	175.0 (1)	C9—C10—C13—C18	−57.7 (2)
O1—C7—C12—C11	−174.6 (1)	C11—C10—C13—C14	−111.7 (2)
C1—Si1—C3—C4	172.7 (1)	C11—C10—C13—C18	124.0 (2)
C1—Si1—C3—C5	−67.7 (1)	C8—C9—C10—C13	−177.9 (1)
C1—Si1—C3—C6	53.3 (1)	C12—C11—C10—C13	178.2 (1)
C2—Si1—C3—C4	−63.1 (1)	C13—C14—C15—C16	52.9 (2)
C2—Si1—C3—C5	56.5 (1)	C14—C15—C16—C17	−51.4 (2)
C2—Si1—C3—C6	177.5 (1)	C15—C16—C17—C18	52.0 (2)
C1—Si1—O1—C7	62.5 (1)	C16—C17—C18—C13	−54.1 (2)
C2—Si1—O1—C7	−57.2 (1)	C17—C18—C13—C14	56.7 (2)
C3—Si1—O1—C7	−177.40 (14)	C18—C13—C14—C15	−56.2 (2)
Si1—O1—C7—C8	95.0 (2)	O2—C16—C17—C18	−82.4 (2)
Si1—O1—C7—C12	−89.6 (2)	O2—C16—C15—C14	83.1 (2)
C7—C8—C9—C10	−0.2 (2)	C19—C16—C17—C18	−149.9 (1)
C8—C9—C10—C11	0.5 (2)	C19—C16—C15—C14	150.5 (1)
C9—C10—C11—C12	−0.2 (2)	C10—C13—C14—C15	177.9 (1)
C9—C8—C7—C12	−0.5 (2)	C10—C13—C18—C17	−178.07 (13)

### 6-[4-(tert-Butyldimethylsilyloxy)phenyl]-1-oxaspiro[2.5]heptane Hydrogen-bond geometry (Å, º)

**Table:**

*D*—H···*A*	*D*—H	H···*A*	*D*···*A*	*D*—H···*A*
C15—H15*A*···O2^i^	0.99	2.54	3.487 (2)	159

Symmetry code: (i) −*x*+3, −*y*+1, −*z*+2.
